# Comparison of the knee joint reaction force between individuals with and without acute anterior cruciate ligament rupture during walking

**DOI:** 10.1186/s13018-022-03136-y

**Published:** 2022-05-03

**Authors:** Hossein Akbari Aghdam, Farzaneh Haghighat, Mohammad Reza Rezaee, Mahsa Kavyani, Mohammad Taghi Karimi

**Affiliations:** 1 Department of Orthopedic Surgery, School of Medicine, Isfahan University of Medical Sciences, Isfahan, Iran; 2 Rehabilitation Sciences Research Center, Shiraz University of Medical Sciences, Shiraz, Iran; 3 Musculoskeletal Research Center, Isfahan University of Medical Sciences, Isfahan, Iran

**Keywords:** Anterior cruciate ligament rupture, Kinematics, Kinetics, Joint reaction force, Muscle force

## Abstract

**Background:**

Anterior cruciate ligament plays a significant role in knee joint stability. It is claimed that the incidence of knee osteoarthritis increases in individuals with anterior cruciate ligament (ACL) rupture. The aim of this study was to evaluate the knee joints reaction force in ACL rupture group compared to normal subjects.

**Method:**

Fifteen patients with acute ACL rupture and 15 healthy subjects participated in this study. The ground reaction force (GRF) and kinematic data were collected at a sampling rate of 120 Hz during level-ground walking. Spatiotemporal parameters, joint angles, muscle forces and moments, and joint reaction force (JRF) of lower extremity were analyzed by OpenSIM software.

**Results:**

The hip, knee and ankle joints reaction force at loading response and push-off intervals of the stance phase during walking was significantly higher in individuals with ACL rupture compared to healthy controls (*p* value < 0.05). Walking velocity (*p* value < 0.001), knee (*p* value = 0.065) and ankle (*p* value = 0.001) range of motion in the sagittal plane were significantly lower in the patients with ACL rupture compared to healthy subjects. The mean value of vertical GRF in the mid-stance, the peak of the hip adduction moment in loading response and push-off phases, the hip abductor, knee flexor and vastus intermedius part of quadriceps muscle forces were significantly higher compared to healthy subjects (*p* < 0.05) while vastus medialis and vastus lateralis produced significantly lower force (*p* < 0.001).

**Conclusions:**

Based on results of this study, lower limb JRF was higher in those with ACL rupture compared to healthy subjects may be due to the compensatory mechanisms used by this group of subjects. An increase in knee JRF in patients with ACL rupture may be the reason for the high incidence of knee OA.

## Introduction

Knee joint stability is achieved by a complex structure consisting of ligaments, joint capsules, and muscles [1]. The anterior cruciate ligament (ACL) is one of the knee joint structures which plays a significant role in this regard. The loss of ACL causes excessive anterior tibia translation relative to the femur [1, 2]. The injuries of ACL are most common in sport-related activities with an incidence of more than 100,000 annual cases in the USA [3].

Deficiency in the performance of ACL should be compensated by the strong contraction of hamstring (which pulls tibia posteriorly). Moreover, some patients prefer to walk with a weaker contraction of quadriceps [4, 5].

It has been shown that reduced knee flexion, internal tibia torsion, and increased knee adduction moment during level walking are the main changes that occurred in the gait of those with ACL injuries [6–8]. It is assumed that gait adaptation in the sagittal plane can lead to knee joint overloading which results in osteoarthritis initiation and progression [9]. This may be due to coping strategy used by this group of the subjects while walking (mainly quadriceps avoidance strategy). Use of this mechanism is achieved by hamstring muscles which finally increase joint loading and will increase the incidence of knee osteoarthritis [10, 11]. There are some studies evaluated the kinetic and kinematic parameters of knee joint of those with ACL injuries and also with ACL reconstruction in normal walking, crossover and pivoting jump and also in single leg vertical drop jump test [12–14]. The results of the study done by Ferrer et al. [13] showed that there was no significant difference between transverse plane kinetic and kinematic parameters in crossover and pivoting jump of those with ACL deficiency and healthy subjects. However, the difference between maximum knee valgus angle and minimum knee flexion angle during single leg vertical drop jump test of those with ACL injuries and healthy football player was significant [12].

Based on the results of various studies, the incidence of knee OA is high after ACL rupture (ACL rupture) [15–17]. This may be due to excessive force applied on the knee joint or maybe due to the compensatory mechanism employed by the subjects. It is also mentioned that excessive tibia torsion may be an abnormal movement mechanism that increases soft tissue degeneration [18].

Increased joint loading has been mentioned as the main reason for degenerative changes after ACL rupture. Joint loading has been evaluated based on the magnitude of the moments applied to the knee joint or based on the use of the modeling approach (Anybody and OpenSIM) or based on the direct approach (use of especial sensors inside of arthroplasty knee joint which measure the loads applied on knee joint during daily activities) [19, 20]. Results of various studies showed that decreased adduction moments are usually seen in the subjects with ACL rupture [6, 21, 22]. The results of a study done by Wellsandt et al. [15] showed that the patients with knee OA have lower reaction force in the involved knee relative to the uninvolved one before and 6 months after ACL rupture.

It seems that although ACL ruptured subjects use both hamstring facilitation and quadriceps avoidance mechanisms to control anterior tibia translation, both forms of muscle compensations alter the distribution of load across tibiofemoral joint [23].

Based on the available studies, it is controversial whether joint loading increases in those with ACL rupture or not (as most of the studies only used the adduction moment as a surrogate for knee loading). Therefore, this study aimed to evaluate the knee joint reaction force in those with ACL rupture compared to normal subjects. The main hypothesis associated with this study was that joint loading increased in the ACL rupture group compared to normal subjects.

## Method

### Participants

Fifteen subjects with ACL rupture (14 male and 1 female) with mean age, height, and weight of 30.5(± 4.6) years, 175.5(± 4.82) cm, and 72.5(± 7.4) kg, respectively, and 15 healthy subjects as a control group (with mean age, height, and weight of 31.4(± 3.6) years, 172.5(± 4.8) cm, and 71.5(± 6.4) kg, respectively) were enrolled in this study. The main criteria to select the subjects were as follows: (1) patients who were in the acute phase after an ACL rupture (less than 6 months), (2) patients with unilateral ACL rupture and (3) without any other musculoskeletal disorders which influences their abilities to stand and walk.

Ethical approval was obtained from the ethical committee of Isfahan University of Medical Science. Moreover, each subject was asked to sign a consent form before participation in the study.

*Data collection* A motion capture system with 7 high-speed cameras (Qualysis, Switzerland) in addition to a Kistler force plate (Kistler Company, USA) was used to collect the three-dimensional motion data of the body segments during walking and the force applied on the foot, respectively. Reflective markers were attached on the first and fifth metatarsal heads, medial and lateral malleolus, heel, medial and lateral knee, greater trochanter, anterior superior iliac spine (ASIS), posterior superior iliac spine (PSIS), and acromioclavicular joint in both right and left sides. Moreover, two reflective markers were attached to the sternum and C7. Subjects were asked to walk with their comfortable speed along the walkway in the gait laboratory (5-m-long) to collect 5 successful trials (trials with complete contact of the foot on the force plate). The data were collected for both sound and injured sides in ACL group and right and left sides in control subjects. The data of both camera and force plate were recorded with sampling rate of 120 Hz.

### Data analysis

Data were filtered with fourth-order Butterworth low-pass filter with 10 Hz cutoff frequency [24]. The global coordinate system of the laboratory (YZX) was then transformed into the OpenSim global system (XYZ) in a Graphical User Interface (GUI) (version 3.3). OpenSIM analysis was done based on use of Rajagopal model. This model was developed based on previous models such as 2354, 2395, and Arnold medals. This model is based on experimental measurement of 21 cadaver lower limbs and MRI of 24 young adult subjects. In this model, a knee model was developed which accurately represents knee joint internal forces. The model was actuated by 80 muscles tendon in lower body and 17 torque actuators. The main advantages of this model compared to other available models include: muscles geometry compared with experimental parameters, maximum isometric force and optimal fiber length are based on comprehensive studies timing of muscles activities matched well with EMG, and was developed based on MRI data collected from healthy young adults [25]. A generic model (Rajagopal) was scaled linearly based on the anthropometric data and also the distances between anatomical markers of static trial for each subject. Then, the joint angles (Inverse Kinematic (IK) tool), joint moments (Inverse Dynamic (ID) tool), muscle excitation (Computed Muscle Control (CMC) tool), Muscle force and joints reaction force (Analyze tool) were analyzed in this study. Actually, there are three approaches to predict muscle forces, including static optimization, computer muscle control (CMC), and neuromuscular tracking (NMT). CMC and NMT are the two most recent used methods based on forward dynamic stimulation. Based on the results of the study done by Lin et al. [26], there is no difference between the results of muscle forces based on these three approaches.

The parameters of interest including the temporospatial parameters (stance phase time and percentage, walking cycle time, stride length, gait velocity, and cadence), GRF parameters in stance phase (maximum of braking and propulsive forces, first (loading response) and second (push-off) vertical GRF in addition to vertical GRF at mid-stance and medial GRF, joint ROM in the whole cycle [lumbar, pelvis, hip, knee (only flex/ext) and ankle (only flex/ext)], maximum joint moments in stance phase (flexion/extension moments of hip, knee, and ankle joints in addition to first and second peaks of hip adduction and hip rotation moments), maximum JRF in stance phase (hip, knee, and ankle anterior/posterior, first and second vertical, and lateral JRF), and maximum muscles force in stance phase (hip abductor, knee flexor and knee extensor muscles) were extracted from the OpenSim results. It should be mentioned that the joint moments were normalized to body mass and the GRF, JRF, and muscles forces were normalized to the body weight of each subject.

The normal distribution of the parameters was checked by the Shapiro–Wilk test. After ensuring that the data followed a normal distribution, the differences between the mean values of the abovementioned parameters between the ACL rupture group and healthy subjects were evaluated by Independent *T* test and the critical alpha was set at 0.05.

## Results

The mean values of the spatiotemporal gait parameter of both healthy and ACL rupture groups are shown in Table 1. The mean value of stride length of those with ACL rupture was 1.23(± 0.155) m compared to 1.38(± 0.4) m for normal subjects (*p* value = 0.000). There was no significant difference in the percentage of the stance phase between healthy subjects and those with ACL rupture (*p *value = 0.093) but both stance time (*p* value = 0.044) and cycle time (*p* value = 0.021) were significantly higher in ACL rupture compared to the control group. Patients with ACL rupture walked with significantly lower velocity (*p* value < 0.001) and cadence (*p* value = 0.001) compared to healthy subjects.

**Table 1:** Spatiotemporal Gait parameters during walking in ACL rupture and healthy groups

Parameters	ACL rupture (mean ± SD)	Normal (mean ± SD)	*p* value
Gait cycle time (s)	1.339 (0.27)	1.098 (0.07)	*0.021*
Stride length (m)	1.233 (0.15)	1.384 (0.14)	*0.016*
Stance time (s)	0.818 (0.24)	0.636 (0.05)	*0.044*
Velocity (m/s)	0.963 (0.26)	1.268 (0.14)	*0.000*
Cadence (step/min)	92.44 (15.97)	109.95 (8.56)	*0.001*
Stance percentage (%)	60.36 (5.11)	57.99 (2.08)	0.093

As can be seen from Table 2, both breaking (*p* value = 0.279) and propulsive (*p* value = 0.007) components of GRF were significantly lower in ACL rupture compared to the control group. Although the mean value of vertical GRF at mid-stance in ACL rupture group was significantly higher than healthy group (*p* value = 0.039), the peak of GRF in loading response (*p* value = 0.93) and push-off (*p* value = 0.226) and also medial GRF (*p* value = 0.993) were not significantly different between the groups.

**Table 2:** Normalized Ground reaction force parameters during walking in ACL rupture and healthy groups

Parameters	ACL rupture (mean ± SD) % of body weight	Normal (mean ± SD) % of body weight	*p* value
GRF-FX1	8.2 (13.4)	13.35 (6.7)	0.279
GRF-FX2	16.98 (2.1)	21.1 (3.7)	*0.007*
GRF-FY1	107.34 (15.92)	106.88 (7.3)	0.932
GRF-FY2	87.47 (16.3)	74.77 (7.2)	*0.039*
GRF-FY3	106.76 (15.7)	113.45 (6.0)	0.226
GRF-FZ	5.66 (3.8)	5.65 (1.6)	0.993

*GRF* ground reaction force, *FX* anteroposterior force, *FY* vertical force, *FZ* mediolateral force, *FX* anteroposterior force, *FY* vertical force, *FZ* mediolateral force

The range of lumbar, pelvic, hip, trunk, knee, and ankle joint motions are summarized in Table 3. The lumbar bending ROM in ACL rupture was 16.7(± 12.7) degree compared to 9.18(± 6.11) in normal subjects (*p* value = 0.043). However, lumbar rotation decreased significantly in ACL rupture compared to control group (*p* value = 0.005). The range of pelvic tilt in the ACL rupture group was more than twice compared to normal subjects (*p* value = 0.021). Although hip joint ROM in the sagittal plane decreased (*p* value = 0.041), hip joint ROM in frontal plane increased significantly (*p* value = 0.005) in the ACL rupture group compared to the control group. The knee (*p* value = 0.065) and ankle (*p* value = 0.001) joint ROM in the sagittal plane was significantly lower in ACL rupture compared to healthy subjects.

**Table 3:** Joint range of motion during walking in ACL rupture and healthy groups

Parameters (in °)	ACL rupture (mean ± SD)	Normal (mean ± SD)	*p* value
Pelvic tilt	12.194 (9.74)	5.533 (4.71)	*0.021*
Pelvic list	17.71 (10.59)	10.251 (3.27)	0.055
Pelvic rotation	16.259 (7.4)	15.474 (6.68)	0.777
Hip flexion/extension	39.329 (10.61)	46.657 (7.38)	*0.041*
Hip abduction/adduction	22.488 (9.48)	14.948 (3.46)	*0.005*
Hip rotation	11.704 (3.85)	19.239 (6.35)	*0.002*
Knee flexion/extension	56.244 (14.49)	63.875 (6.59)	*0.065*
Ankle dorsi/plantar flexion	25.77 (4.7)	31.98 (4.18)	*0.001*
Lumbar flexion/extension	16.727 (12.72)	9.179 (6.11)	*0.043*
Lumbar lateral bending	16.328 (7.03)	13.077 (4.2)	0.136
Lumbar rotation	23.467 (8.59)	37.849 (13.37)	*0.005*

The mean values of hip joint flexion/extension, adduction/abduction, internal/external rotation moments, as well as knee flexion/extension and ankle dorsi/plantar flexion of ACL rupture group and normal subjects, are reported in Table 4. The hip extension moment was significantly lower in ACL rupture than control group (*p* value < 0.001). The peaks of the hip adduction moment in loading response (*p* value = 0.302) and push-off intervals of stance phase in the ACL rupture group were higher than those in the healthy control group (*p* value = 0.079). The knee extension moment in mid-stance was lower in ACL rupture compared to healthy group (*p* value = 0.001).

**Table 4:** Normalized joint moments during walking in ACL rupture and healthy groups

Parameters (Nm/BM)	ACL rupture (mean ± SD)	Normal (mean ± SD)	*p* value
Hip flexion	0.657 (0.18)	0.536 (0.18)	0.11
Hip extension	0.436 (0.15)	1.044 (0.3)	*0.000*
Hip adduction, first peak	0.636 (0.35)	0.508 (0.15)	0.302
Hip adduction, second peak	0.767 (0.32)	0.586 (0.19)	0.079
Hip rotation	0.077 (0.07)	0.081 (0.03)	0.875
Knee flexion	0.166 (0.09)	0.175 (0.08)	0.822
Knee extension	0.421 (0.11)	0.709 (0.3)	*0.001*
Knee flexion	0.284 (0.23)	0.148 (0.09)	0.111
Ankle plantar flexion	0.213 (0.07)	0.311 (0.08)	*0.007*
Ankle dorsi flexion	1.518 (0.21)	1.417 (0.2)	0.232

**Fig. 1:**
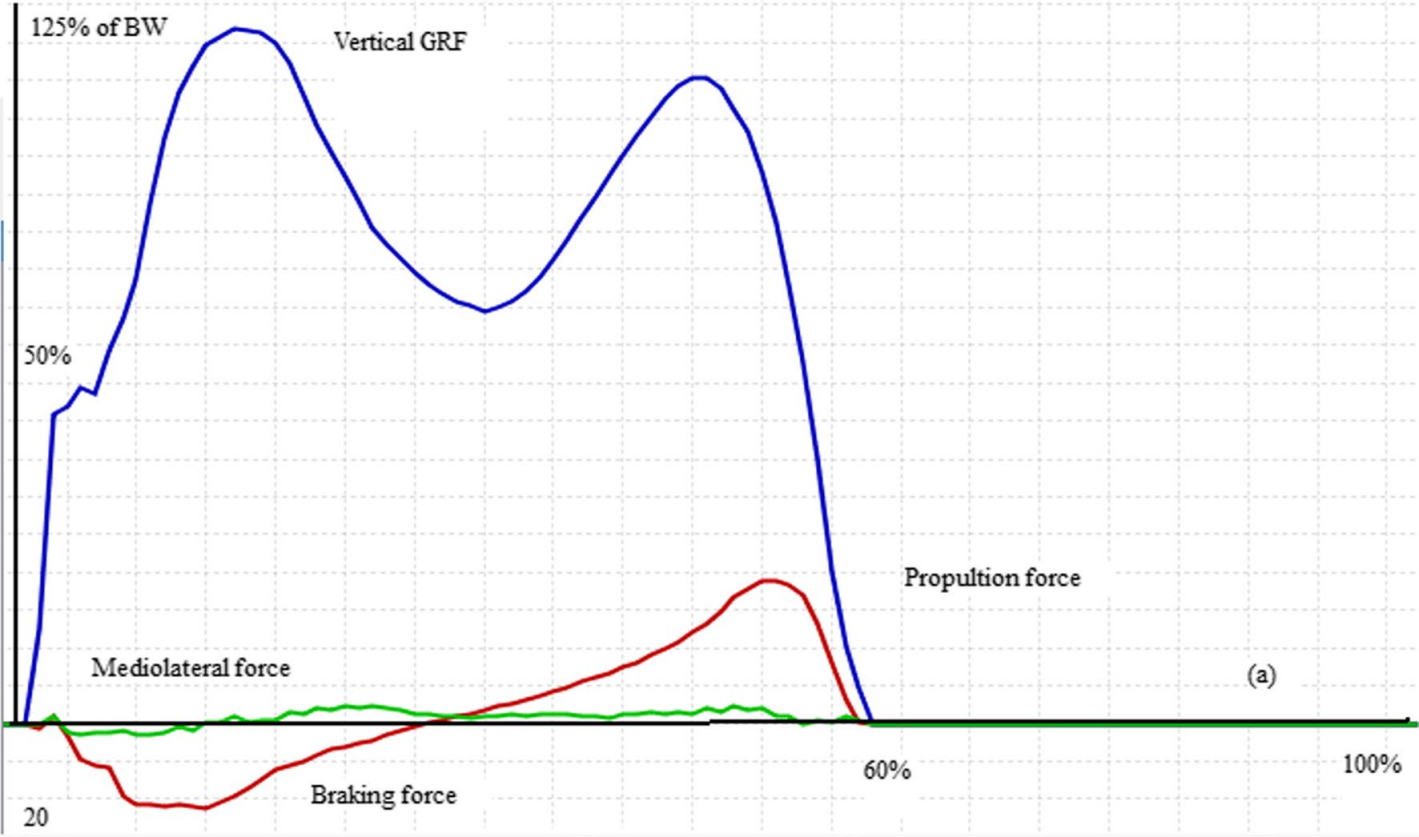
Ground reaction force applied on the leg of a subject with ACLR

The peak of hip, knee, and ankle JRF in 3 axes are summarized in Table 5 and Fig. 1. As can be seen, vertical hip JRF at loading response (*p* value = 0.002) and also hip joint lateral shear force (*p* value = 0.05) were significantly higher in the ACL rupture than healthy group. Regarding the knee joint, the posterior shear force was significantly lower, and the anterior shear force (*p* value < 0.001) and the vertical knee JRF at push-off (*p* value = 0.011) were significantly higher in ACL rupture compared to healthy control group. The between-group differences in vertical knee JRF at loading response (*p* value = 0.718) and lateral shear force (*p* value = 0.0.688) were not significant. Vertical ankle joint reaction force at both loading response (*p* value < 0.001) and push-off (*p* value = 0.001) was significantly higher in patients with ACL rupture compared to healthy ones while the differences in ankle anterior (*p* value = 0.0.176), posterior (*p* value = 0.0.69) and lateral (*p* value = 0.0.58) shear forces were not significant between groups. It should be noted that global coordination system used in OpenSIM is the same for force plate, and bony components, tibia, femur (X is anteroposterior, Y is vertical and Z is mediolateral direction).

**Table 5:** Normalized joint reaction forces during walking in ACL rupture and healthy groups

Parameters (N/BW)	ACL rupture (mean ± SD)	Normal (mean ± SD)	*p* value
Hip FX1	1.378 (0.8)	0.892 (0.42)	0.1
Hip FX2	2.867 (0.98)	3.172 (0.91)	0.417
Hip FY1	5.58 (1.75)	3.67 (1.09)	*0.002*
Hip FY2	6.302 (2.54)	5.377 (1.23)	0.302
Hip FZ	1.777 (0.99)	1.046 (0.45)	*0.05*
Knee FX1	1.176 (0.38)	0.432 (0.32)	*0.000*
Knee FX2	1.37 (0.5)	2.615 (0.37)	*0.000*
Knee FY1	4.2 (1.15)	4.04 (1.08)	0.718
Knee FY2	5.206 (1.65)	3.541 (0.45)	*0.011*
Knee FZ	0.388 (0.17)	0.365 (0.12)	0.688
Ankle FX1	1.155 (0.59)	0.874 (0.21)	0.176
Ankle FX2	3.201 (0.91)	2.589 (0.76)	0.069
Ankle FY1	6.552 (1.22)	2.957 (0.69)	*0.000*
Ankle FY2	7.84 (1.83)	4.894 (0.66)	*0.001*
Ankle FZ	0.404 (0.19)	0.367 (0.06)	0.58

**Fig. 2:**
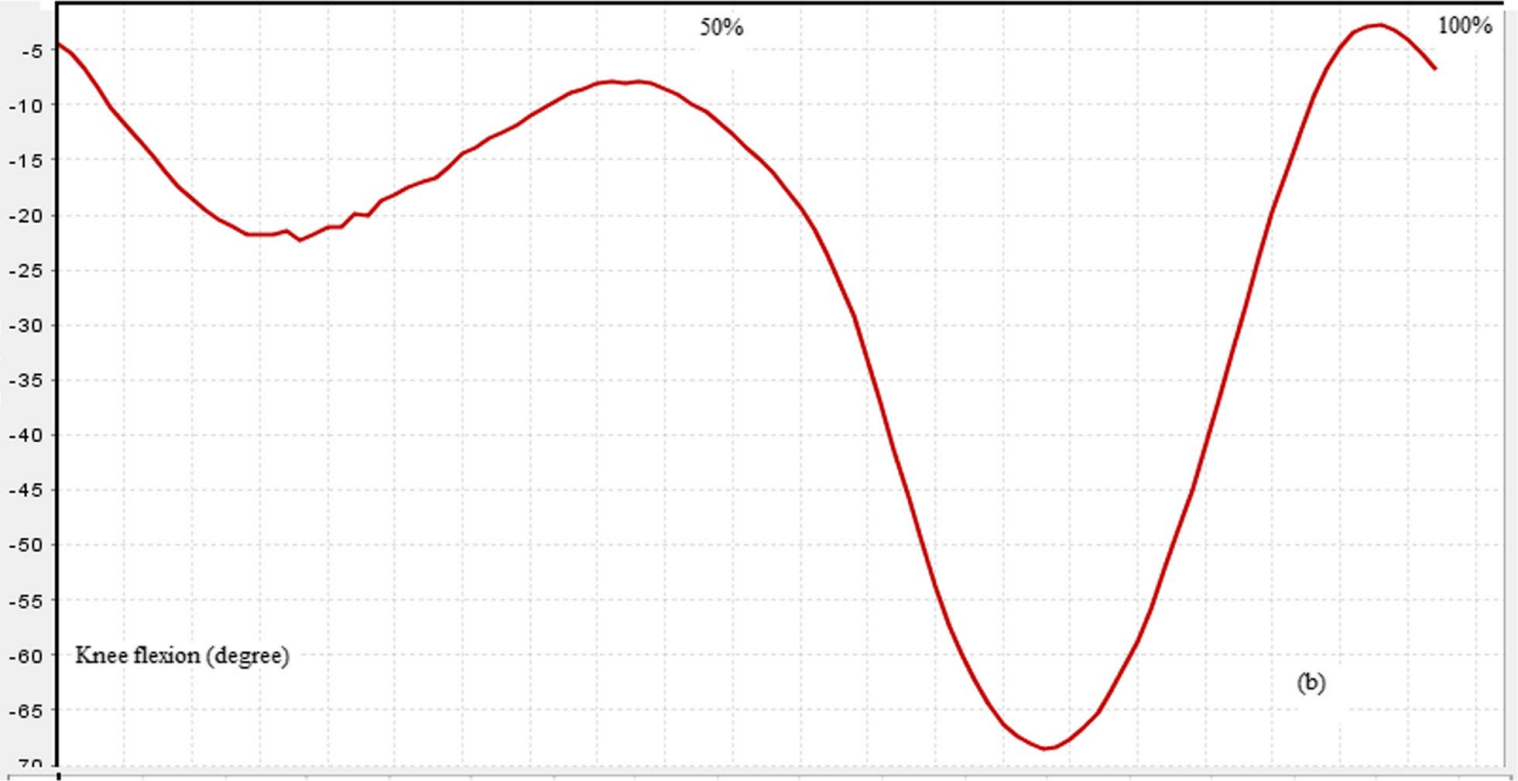
Knee joint flexion/extension in walking of a subject with ACLR

**Fig. 3:**
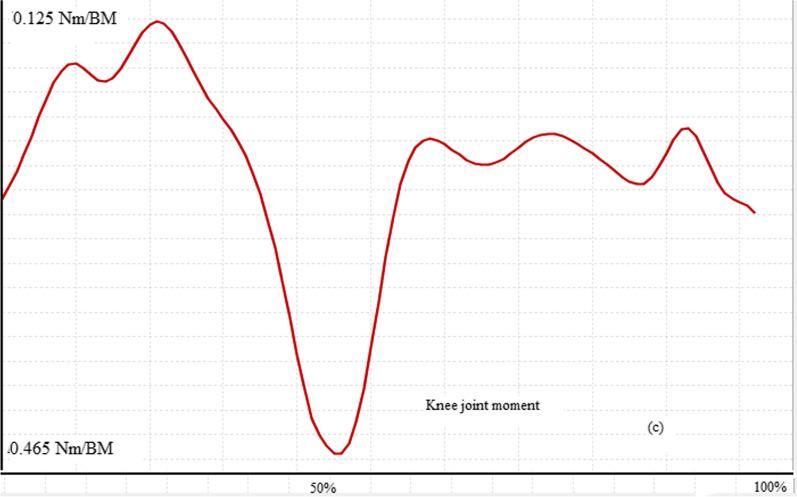
Moment of knee joint in sagittal plane of a subject with ACLR

**Fig. 4:**
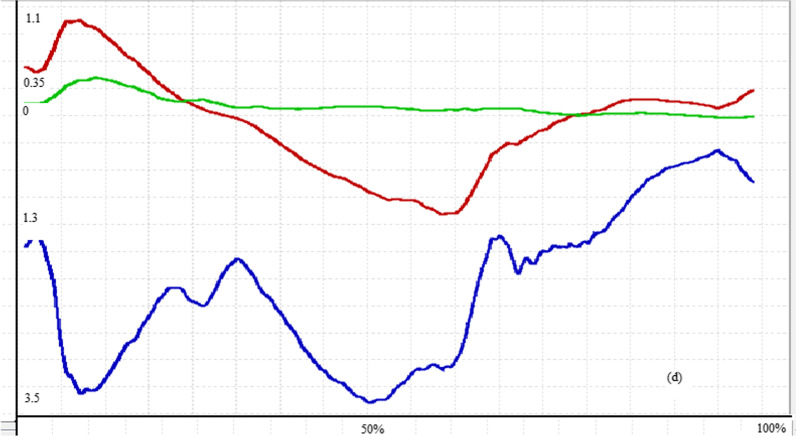
Knee joint contact force of a subject with ACLR, (N/BW)

The maximum force generated by lower limb muscles of both normal and those with ACL rupture is presented in Table 6. As can be seen from this table, the middle fibers (*p* value < 0.001) and posterior fibers (*p* value < 0.001) of gluteus medius muscle and also knee flexors including Semimembranosus (*p* value = 0.027), long (*p* value = 0.002) and short (*p* value < 0.001) head of Biceps femoris generated significantly higher force in ACL rupture compared to healthy subjects. Vastus medialis (*p* value < 0.001) and vastus lateralis (*p* value < 0.001) parts of quadriceps muscle produced significantly lower while the vastus intermedius (*p* value = 0.01) produced significantly higher force in ACL rupture than healthy group. Other muscles did not show any significant differences between study groups. Figures 1, 2, 3 and 4 show the pattern of ground reaction force, flexion/extension moment of knee joint in sagittal plane and joint contact force of knee joint, respectively.

**Table 6:** Normalized muscle forces during walking in ACL rupture and healthy groups

Muscles (N/BW)	ACL rupture (mean ± SD)	Normal (mean ± SD)	*p* value
Gluteus medius (anterior)	0.982 (0.294)	0.823 (0.243)	0.141
Gluteus medius (middle)	0.592 (0.233)	0.186 (0.044)	< *0.001*
Gluteus medius (posterior)	0.66 (0.27)	0.21 (0.109)	< *0.001*
Rectus femoris	1.084 (0.532)	1.33 (0.518)	0.249
Vastus medialis	0.207 (0.059)	0.43 (0.14)	< *0.001*
Vastus intermedius	0.258 (0.085)	0.185 (0.053)	*0.01*
Vastus lateralis	0.441 (0.137)	1.118 (0.521)	< *0.001*
Semimembranosus	0.808 (0.27)	0.567 (0.25)	*0.027*
Semitendinosus	0.279 (0.137)	0.319 (0.09)	0.364
Biceps femoris (long head)	0.559 (0.232)	0.345 (0.089)	*0.002*
Biceps femoris (short head)	0.577 (0.269)	0.22 (0.051)	< *0.001*

## Discussion

This study aimed at evaluating articular loading in ACL rupture patients using OpenSIM software extracting joint reaction force based on kinematic data, external loads, and muscle forces. Although joint reaction force is a basic feature of joint loading, it has remained largely unknown for ACL rupture patients.

Based on spatiotemporal findings (Table 1), the ACL rupture group exhibited shorter step length and slower walking speed compared to the healthy individuals. This finding is consistent with the findings of previous studies [27–29]. The shorter step length of the ACL rupture subjects is because these patients do not fully extend their injured knees during the stance phase and at the terminal swing and it is suggested that this is due to the functional deficit (instability of knee joint mostly in anteroposterior plane) in the ACL rupture patients. An intact ACL restrains anterior translation of the tibia when the knee approaches full extension, and after ACL rupture, patients use the adaptation strategy of limiting knee extension to avoid knee joint instability [27].

Slower walking speed and longer stance time in the ACL rupture group may be due to their fear of further injury and less confidence in their injured knee. By adopting this strategy, they seem to try to control the motions of the knee joint, decrease the loads applied to the joint, and improve dynamic stability [30], whether this strategy is successful to reduce joint reaction force or not is the question that is addressed below.

**Fig. 5:**
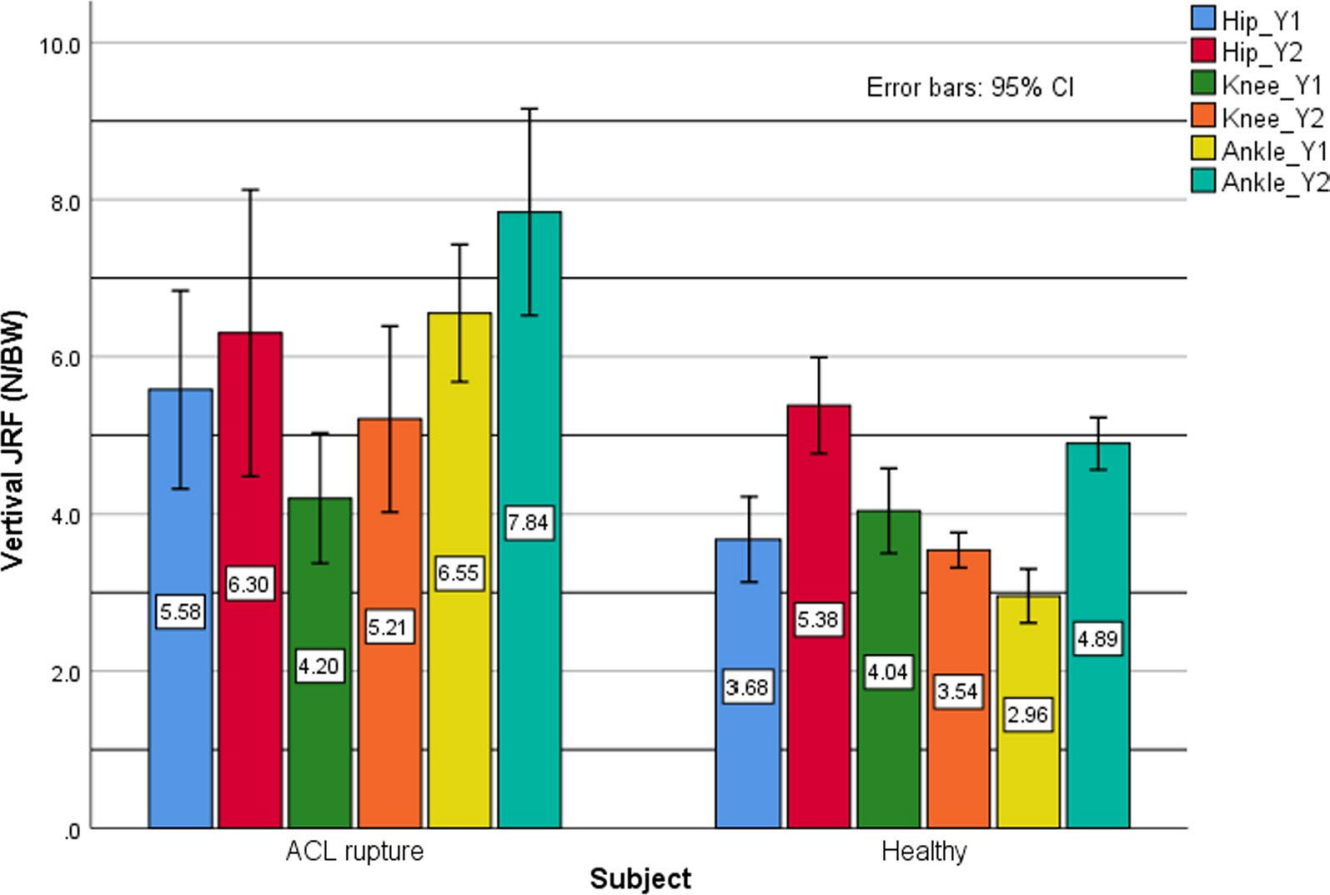
The comparison of the hip, knee, and ankle joints reaction force at loading response (Y1) and push-off (Y2) phases during walking between ACL rupture and healthy subjects

Based on JRF results in Table 5, significantly greater anterior shear force and lesser posterior shear force in addition to greater vertical force during push-off were observed in ACL rupture patients compared to the control group. Significantly greater vertical force at loading response and more medial shear force were also seen in the hip joint in ACL rupture patients (Fig. 5). All ankle JRFs in the ACL rupture group were more than healthy subjects but only the vertical forces were statistically meaningful. Altered joint loading has an important role in the development of degenerative changes among individuals with ACL rupture. Reaction forces and shear forces are of important loading features [31]. Overall, based on the results of this part of the research, it can be concluded that JRF increased in the ACL rupture group compared to controls. Overloading of joint contact areas, which normally are unloaded, is thought to be the reason for early-stage degeneration in ACL rupture knees [32]. Also based on animal models, it was found that cartilage health suffers from repetitive overloading [33]. Moreover, since the knee adduction moment has been proposed as an indirect estimate of the joint loading, higher knee joint loading in the current study confirms the results of previous studies that reported a positive correlation between knee adduction moment and the severity and progression of knee OA [34, 35]. However, this finding is in contrast with those of Gardinier et al. [31] and Khandha et al. [36] who reported that the tibiofemoral reaction force is significantly lower in ACL rupture knees compared to the uninjured knees [31, 36] and also control group [36]. It is well known that the dominant hypothesis about the mechanism responsible for cartilage degeneration is excessive joint loading and elevated stress [37]. The controversy between the finding of the current study and the studies by Gardinier [31] and Khandha [36] could arise from different methodologies used to compute the joint reaction force. In these studies, the JRF is calculated using the EMG-driven model while in the current study, the joint reaction force is extracted from the OpenSIM model based on computer muscle control [25]. However, due to the paucity of studies regarding joint loading calculation in ACL rupture patients, more researches with the same methodology are needed to make a conclusion.

In the research done by Khandha et al., 36 subjects with ACL injuries were compared with 12 normal subjects, with age of 29 and 23 years, respectively. The knee joint contact force was determined based on use of EMG data collected from medial and lateral hamstring, medial and lateral gastrocnemius, rectus femoris and medial and lateral vasti muscles. There was no significant difference between the walking speed of those with ACL injuries and normal subjects (walking speed between 1.56 and 1.57 m/s). They concluded that contracture of the muscles did not increase knee joint contact force in ACL injuries subjects. However, it should be noted that the subjects participated in their study did not use any compensatory mechanism, as there was no difference between walking speed of normal and those with ACL injuries. Moreover, there was no significant difference between the peak of knee flexion and knee adduction between the groups. The other important point was that knee joint contact force was calculated based on limit number of muscles and the performance of some muscles such as soleus was ignored (although this muscles control the ankle joint motion, it also control the motion of tibia which indirectly stabilize the knee joint). It seems that in the research done by Khandha et al., no matching was done between normal and those with ACL injuries. In contrast in the current study, the walking speed of the ACLR group was significantly less than that of normal subjects, which confirmed that they used a compensatory mechanism to control the motion of knee joint.

In the research done by Gardinier et al., the loads applied on knee joint in ACL injuries side were compared with that of contralateral side (there was no control group). The same method as the research of Khandha was used to determine knee joint contact force. The subjects walked with speed of 1.53 m/s, which again confirmed that the subjects did not use any compensatory mechanism to control the motions. Another important point was that the effects of ACL rupture not only influence the motion of knee joint in the injured sides but also could influence the motions of other joints of body. Therefore, comparison the injured side and sound side seems to be not too practical.

ACL rupture patients commonly use some compensation strategies including altered sagittal knee excursions and moments, altered GRF as well as elevated muscular co-activation, early after the injury to decrease joint instability which may contribute to the altered knee joint loading. Hence, in the second part of this research, we aimed at evaluating common compensatory strategies after the ACL rupture.

As mentioned above, GRF data in Table 2 show that the only greater value in ACL rupture knees was vertical ground reaction force during mid-stance, so generally it seems that ACL rupture patients walked more slowly to reduce GRF on the injured knee compared with the control group. However, higher vertical GRF during mid-stance tends to flex the knee, so it needs the higher activation of the quadriceps to counteract. On the other hand, the ACL rupture patients try not to activate the quadriceps muscle to avoid the anterior translation of the tibia and resultant instability. According to Table 5, these patients have a significantly higher activity of vastus intermedius but no other heads of quadriceps. It seems they attempted to balance between reducing quadriceps activity and counteracting the higher vertical GRF during mid-stance.

Compared to the healthy control group, hamstring activity was significantly higher in ACL rupture patients and Quadriceps muscle activity was lower in vastus medialis and vastus lateralis parts (Table 6). Although lower activity from vastus medialis and vastus lateralis partially confirms the quadriceps avoidance strategy which aimed at avoiding knee instability [38], higher activity from vastus intermedius plus higher hamstring activity could suggest some co-contraction among hamstring and quadriceps muscles which have the same purpose as quadriceps avoidance strategy and is formed to stabilize the knee in the absence of passive ligamentous restraint [39–42].

The theoretical reason for the quadriceps avoidance strategy is that the knee extensors are considered as ACL antagonists because the quadriceps muscle contraction draws the tibia anteriorly when the knee joint is near full extension [43].

Higher activity of hamstring complex is because hamstring muscle tendons attach on the proximal tibia and its contraction, exert a posterior force on the tibia, so considered as an ACL agonists [43]. This hamstring facilitation strategy in addition to the quadriceps avoidance strategy could be attributed to the lower knee extension moment in the ACL rupture group which is consistent with previous studies [39, 40].

Overall, lower sagittal plane knee range of motion (Table 3), hamstring facilitation strategy, quadriceps avoidance strategy, and also lower knee extension moment may be suggestive of a stabilization strategy that is commonly seen in ACL rupture patients.

Higher activity of hip abductor muscle (all parts of gluteus medius) (Table 6) and also higher hip adduction moment (Table 4) which were seen in the current study can be related. Ipsilateral trunk lean and pelvis tilt, which are considered as two common mechanisms of ACL rupture, occur due to weak hip abductors to reduce the demand on these muscles. These aberrant motions can move the resultant GRF vector toward the stance limb and subsequently increase knee valgus angle which can increase knee joint loading [44, 45]. It seems that the ACL rupture subjects try to limit ipsilateral trunk and pelvis motions by increased hip abductor activity. Significantly higher frontal plane pelvis tilt and list and also higher frontal plane lumbar bending (although statistically non-significant) may suggest that this increased activity of commonly weak hip abductor muscles is not enough. The internal response to counterbalance the ipsilateral pelvis tilt and, subsequently, trunk lean is increased hip adduction moment [46] which may be the reason for the observed hip adduction moment trend toward significant values found in the current study.

It should be emphasized that walking speed is an important parameter which influences the joint reaction force [30]. Those with ACL ruptures decrease their walking speed to reduce the JRF. However, based on the results of this study, although those with ACL rupture had a decrease in walking speed (Table 1), their JRF components mostly increased compared to normal subjects (Table 5). Therefore, walking speed is not a confounding factor which influenced the output of this study.

Some limitations should be acknowledged in this study. The OpenSim model used in the current study [25] has only one degree of freedom (DoF) in the knee joint. Although this is a validated model and has been used in several studies, it would be great to develop a new model with 3 DoF in the knee joint to evaluate the adduction\abduction moments as well as internal\external rotation moments of the knee joint. Further studies must be conducted to compare the knee moments in all DOFs and the JRF between ACL rupture subjects and healthy individuals to see how they correlate to each other. Further research also should investigate whether increased joint reaction forces found in the current study, normalize with ligament reconstruction. Lack of EMG of lower limb muscles is another limitation associated with this study.

## Conclusion

ACL rupture patients walked with increased loads on their injured knee in the acute phase after injury compared to healthy controls. Lesser knee extension moment and knee sagittal plane excursion, higher hamstring and intermedius muscles activity, lesser vastus medialis and lateralis activity and also greater hip abductor muscles activity and hip adduction moment also observed in ACL rupture patients that may be suggestive of adopting a stabilization strategy aimed at reducing knee joint instability; these compensations may be attributed to greater knee joint loading in this population of the patients compared to healthy individuals.

## Data Availability

All materials are available.

## Abbreviations

ACL: Anterior cruciate ligament
GRF: Ground reaction force
ASIS: Anterior superior iliac spine
PSIS: Posterior superior iliac spine
GUI: Graphical user interface
ID: Inverse dynamic
CMC: Computer muscle control
NMT: Neuromuscular tracing
ROM: Range of motion
