# Correction: Comparison of the knee joint reaction force between individuals with and without acute anterior cruciate ligament rupture during walking

**DOI:** 10.1186/s13018-022-03182-6

**Published:** 2022-06-03

**Authors:** Hossein Akbari Aghdam, Farzaneh Haghighat, Mohammad Rezaie, Mahsa Kavyani, Mohammad Taghi Karimi

**Affiliations:** 1 Department of Orthopedic Surgery, School of Medicine, Isfahan University of Medical Sciences, Isfahan, Iran; 2 Rehabilitation Sciences Research Center, Shiraz University of Medical Sciences, Shiraz, Iran; 3 Musculoskeletal Research Center, Isfahan University of Medical Sciences, Isfahan, Iran

## Correction to: Journal of Orthopaedic Surgery and Research (2022) 17:250 10.1186/s13018-022-03136-y

Following publication of the original article [1], the authors identified an error in the author’s family name and affiliations of Mohammad Rezaie.

The correct author’s family name and the affiliations are given below:

The author’s family name is “Rezaie” and the affiliation is “Rehabilitation Sciences Research Center, Shiraz University of Medical Sciences, Shiraz, Iran”.

The author group has been updated above, and the original article [1] has been corrected.
